# Prevalence and serological profile of anti-DFS70 positive subjects from a routine ANA cohort

**DOI:** 10.1038/s41598-019-38686-5

**Published:** 2019-02-18

**Authors:** Teresa Carbone, Vito Pafundi, Giuseppina Tramontano, Michele Gilio, Maria Carmela Padula, Angela Anna Padula, Salvatore D’Angelo

**Affiliations:** 1 IReL – Rheumatology Institute of Lucania - San Carlo Hospital, Potenza, Italy; 2 Immunopathology Laboratory, San Carlo Hospital, Potenza, Italy; 3 Basilicata Ricerca Biomedica (BRB) Foundation, Potenza, Italy

## Abstract

Anti-Dense Fine Speckled 70 (DFS70) antibodies are a common finding in clinical laboratory referrals. High prevalence of DFS70 autoantibodies in healthy population and usual negative association with Antinuclear Antibody (ANA)-associated autoimmune rheumatic diseases (AARD) were reported. The aim of this study was to evaluate the prevalence of DFS70 autoantibodies and their association with other autoantibodies in the context of a routine ANA referral cohort. Consecutive sera submitted for ANA screening were analyzed for anti-DFS70 antibodies by indirect immunofluorescence (IIF) (n = 3175, 1030 men and 2145 women) then confirmed by immunoblotting. Anti-DFS70 positive samples were also assayed for a large spectrum of other circulating autoantibodies. The prevalence of anti-DFS70 antibodies was 1.7% in the whole population and 4.6% in the ANA-positive samples. Comparison between DFS70 IIF and immunoblotting showed an excellent correlation between the two methods. The prevalence of anti-DFS70 positive was significantly higher in females (2.1%, 45/2145) than in males (1.0%, 10/1030). Of note, no concomitant autoantibodies were found in the DFS70-positive male group compared with DFS70-positive females group that showed other serum autoantibodies in the 51% of cases. Anti-DFS70 reactivity in male population may represent an useful biomarker predicting the absence of other autoantibodies. On the contrary, the serological profile of DFS70-positive females required further investigations in order to define the presence of concomitant disease-marker autoantibodies.

## Introduction

Antinuclear antibodies (ANAs) detection by indirect immunofluorescence (IIF) represents a sensitive routine method thus recommended as the screening test of choice by a study group of the American College of Rheumatology^1^. The presence of ANAs is a serological hallmark of systemic autoimmune rheumatic diseases, but their presence in sera from healthy people was also reported^2^. Anti-Dense Fine Speckled 70 (DFS70) antibodies, also known as lens epithelium-derived growth factor (LEDGF), were recently identified as associated to a specific ANA IIF pattern characterized by irregularly distributed, fine-granular fluorescence of the nuclei in the interphase and of the metaphase chromatin^3–6^. Current knowledge of the DFS70/LEDGF autoantigen-autoantibody system identifies the antigen as a transcription co-activator able to upregulate some stress protective and inflammatory genes. This function could contribute to the cellular survival under environmental stress factors, both in health and in disease context^7,8^. The alteration of DFS70 function or structure may trigger disease pathogenesis and autoantibody elicitation. The autoantibodies are preferentially of the IgG class and target a conserved region in DFS70 C-terminal domain. The prevalence of anti-DFS70 antibodies was analyzed in different cohorts with value ranged from 0.8% to 16.6%^9–22^. These variations may be related to differences in the patient selection criteria and methodological aspects as different HEp-2 substrates, to inter-reader variability in pattern assignment and variation in screening dilution. Furthermore, in some studies, no confirmatory analyte-specific immunoassays were employed. Also, the agreements between DFS70 IIF suspicion and confirmation by specific assays varied widely among studies. The discrepancies were related to different antigen exposure and selection (full length LEDGF or selected antigenic region), analytical sensitivity/specificity and manufacturer’s cut-off of the various confirmatory assays^23^. However, use of DFS70-specific immunoassays was recently suggested by a study showing a low accuracy in assessing the DFS70 IIF pattern by experienced technologists^24^.

Anti-DFS70 antibodies were initially described in patients with interstitial cystitis and later in heterogeneous chronic inflammatory conditions, tumours and even in apparently healthy individuals, but their clinical impact is still unknown^25–28^. It was reported that none of anti-DFS70 positive subjects showed symptoms suggestive of an AARD after clinical follow-up of 4 years^18^.

Some previous studies suggested that the isolated anti-DFS70 reactivity could be taken as biomarker to exclude AARD (Antinuclear Antibody (ANA)-associated autoimmune rheumatic diseases) from ANA-positive healthy individuals^9,11,29–31^. Understanding the serological and clinical profile of anti-DFS70 positive subjects thus could avoid inappropriate referral to care specialists and follow-up tests for healthy people. Accordingly, the aim of this study was to define the prevalence of anti-DFS70 antibodies in a routine diagnostic laboratory setting and the associated serum autoantibodies to support the clinical use of these markers.

## Methods

### Samples

Collected samples consisted of serum specimens sent to the Immunopathology Laboratory of the San Carlo Hospital of Potenza by outpatients or inpatients (Cardiology, Dermatology, Endocrinology, Geriatrics, Gynaecology, Haematology, Infectious Disease, Internal Medicine, Nephrology, Neurology, Oncology, Ophthalmology, Pneumology and Rheumatology Units) for ANA analysis with IIF. Paediatric subjects were excluded. Confirmed IIF DFS70 positive adult patients were asked to participate in the present study. Recruited patients have given their informed consent. Collection of patient samples and laboratory procedures were carried out according to Common Regional Ethical Committee of Basilicata (CEUR) (Authorization Number: 705/2017).

### ANA Testing

ANA were detected by commercial ANA HEp-2000 Indirect Immunofluorescent assays (HEp-2000 Fluorescent ANA-Ro Test System, Immuno Concepts N.A., Sacramento, CA, USA). ANA Kits were used according to the appropriate manufacturer’s instructions. The screening dilution was 1:160. Automated instrument for IIF preparation (Gemini Combo, Stratec Biomedical) and anti-human IgG specific fluorescein-labelled (FITC) conjugate were used. Subsequently, slides were assessed with a fluorescence microscope. For each well, 4 microscopic fields with 20x objective with Image Navigator (Immuno Concepts N.A., Sacramento, CA, USA) were captured. This instrument is based on a conventional fluorescence microscope with a motorized stage, high-intensity LED light source, digital camera, computer and proprietary software. Manufacturer’ Fluorescence Index (F.I.) cut-off value for HEp-2000 is equal to 30. Positive/negative interpretation based on a F.I. was evaluated during focusing and confirmed by expert-readers. Positive samples were also tested using the conventional serial dilution method (1:160, 1:320, 1:640, 1:1280 until to 1:2560) to assign end-point titer. The titer was defined as the reciprocal of the highest dilution of serum that still shows immunofluorescent staining.

### Immunoblot Anti-DFS70 Assay

The serum samples positive for DFS70-like pattern in IIF were further processed for detection of human IgG autoantibodies against DFS70 specificity (truncated sequence of the DFS70 antigen (residues 349–435)) by Immunoblotting assay. In addition, confirmed anti-DFS70 positive samples, were also evaluated for the antibodies against Sm, U1-RNP, Sm/RNP, SSA/Ro60kD, SSB, Scl-70, PM-Scl 100, Ku, CENP-A/B, PCNA, Mi-2 antigens using immunoblotting kit (Alphadia, Wavre, Belgium) by automatic Blu Diver Instrument (BDI) (Alifax, Polverara (PD), Italy). During the automated test procedure, the BDI sequentially incubates the strips in the wells of ready-to use reagent cartridges. Human antibodies bind the corresponding specific antigen(s) on the membrane and enzyme activity leads to development of purple dots on the membrane pads. The intensity of the coloration is directly proportional to the amount of antibody present in the sample. Semi-quantitative interpretation was done by Dr Dot Software (Alifax) and scanning system using Arbitrary Unit (AU).

### Detection of other autoantibodies

Anti-DFS70 positive samples were tested for the presence of other serum autoantibodies: anti-extractable nuclear antigen (ENA) (RNP/Sm, SSA/Ro, SSB/La, Scl-70, Jo-1, CENP-B), anti-thyroid peroxidase (TPO), anti-thyroglobulin (TGAb) and anti-tissue transglutaminase (tTg)-IgA by a fully automated chemiluminescence analyzer LIAISON XL (Diasorin, Saluggia (VC), Italy) using a ‘Flash’ chemiluminescence technology (CLIA) with paramagnetic microparticle solid phase; anti-dsDNA by the *Crithidia luciliae* IIF Test (Euroimmun, Padova (PD), Italy); IgG class of anti-mitochondrial (AMA), anti-smooth muscle (ASMA), anti-liver/kidney/microsome type 1 (LKM), and anti-parietal cells (PCA) by triple IIF test on rat liver, stomach and kidney substrates (Alphadia); anti-neutrophil cytoplasmic myeloperoxidase (MPO) and proteinase 3 (PR3) through enzyme immunoassay (GA GENERIC assays GmbH, Dahlewitz, Germany) performed on automatic Immunomat Serion ELISA analyzer (SERION Immunologics, Wurzburg, Germany) as recommended by the manufacturer; anti-Saccharomyces Cerevisiae IgG/IgA (ASCAs) and anti-cardiolipin/β2-GPI complex and β2-GPI isolated protein IgG (aCL) using immunodot kit, a previously described diagnostic assay platform.

### Statistical analysis

Statistical analyses were performed using the Graph Pad Prism statistical package (v5; Graph Pad Software, San Diego, CA). Statistical significance was calculated using the non-parametric Mann-Whitney-U test and correlations were analyzed by the Spearman’s rank correlation test and R-squared analyses (R^2^); categorical variables were compared using Fisher’ Test. A *p* value < 0.05 was considered statistically significant.

### Ethics approval and consent to participate

The study was approved by the Regional Ethics Committee (Authorization Number: 705/2017).

## Results

### Prevalence of DFS70 antibodies

**Figure 1:**
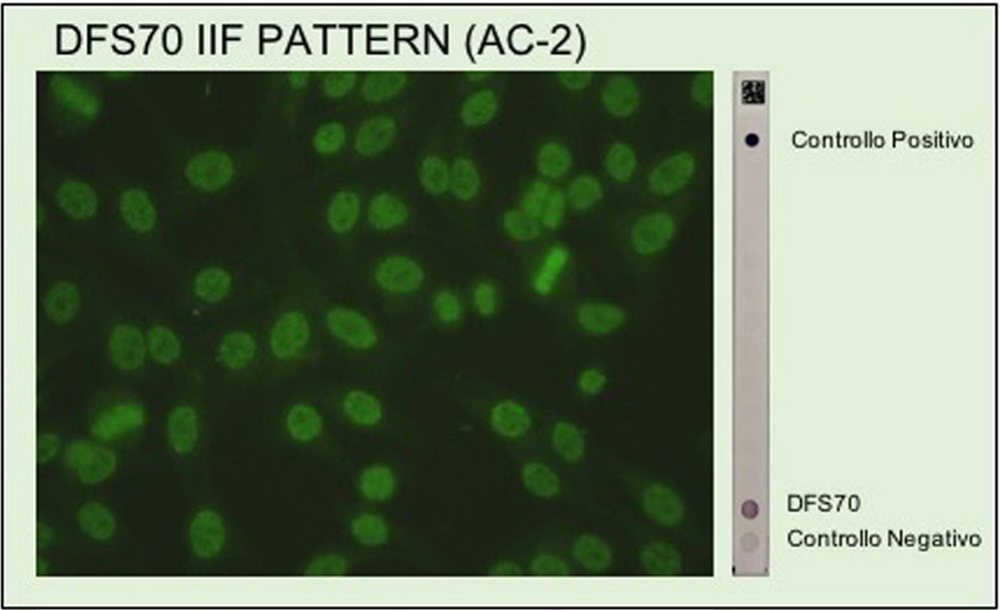
The DFS70 IIF pattern is characterized by staining of dense fine speckled in interphase nuclei and brightly fluorescent mitotic chromosomes. Samples showing a DFS70 IIF pattern were confirmed for specific antibody reactivity using an immunoblotting assay.

Selected cohort included 3175 sera (M = 1030, F = 2145) from consecutive adult subjects submitted for routine ANA testing. Only 45.8% (55/120) of the presumptive DFS70 pattern on the ANA-IIF test were confirmed by immunoblotting, therefore sera negative by dot blot method were included in ANA positive group. Anti-DFS70 antibodies (Fig. 1) were observed in 1.7% (55/3175) of whole series and 4.6% (55/1191) in the ANA-positive samples. The prevalence of anti-DFS70 positive females (2.1%, 45/2145) was statistically significant higher than males (1.0%, 10/1030) (p = 0.02). We found a striking female predominance both in ANA positive group (F/M, 903/288) that in anti-DFS70 positive samples (F/M, 45/10). Comparison among the mean ages of the ANA negative series (51.6 ± 16.8 yrs) versus anti-DFS70 negative/ANA positive group (52.6 ± 17.1 yrs) and anti-DFS70 positive subjects (49.5 ± 15.5 yrs) showed no statistically significant differences (p > 0.05). No statistical difference could be shown between the age in both sexes.

### Analysis of the Referring Physician pattern of samples displaying Dense Fine Speckled pattern

**Figure 2:**
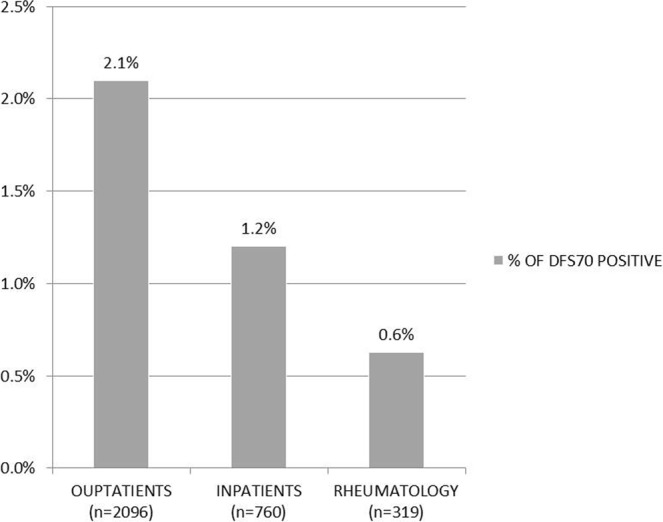
Summary prevalence of anti-DFS70 positive samples obtained from different referring clinical Department that included Rheumatology and inpatients (Cardiology, Dermatology, Endocrinology, Geriatrics, Gynaecology, Haematology, Infection Disease, Internal Medicine, Nephrology, Neurology, Oncology, Ophthalmology, Pneumology) versus outpatients.

Analyzed samples were obtained by outpatients and inpatients coming from various Departments of the San Carlo Hospital of Potenza. About anti-DFS70 positive subjects, 5.3% came from Internal Medicine, 3.6% from Neurology, Haematology and Rheumatology, respectively, 1.8% from Cardiology, 1.8% from Endocrinology and remaining 80.3% from outpatients. By comparative analysis of referring clinical departments emerged that anti-DFS70 autoantibodies were more prevalent in samples from outpatients (2.1%, 44/2096) versus inpatients (1.2%, 9/760) and Rheumatology (0.6%, 2/319) (Fig. 2).

### Quantitative comparison of anti-DFS70 antibodies IIF titers and immunoblotting

**Figure 3:**
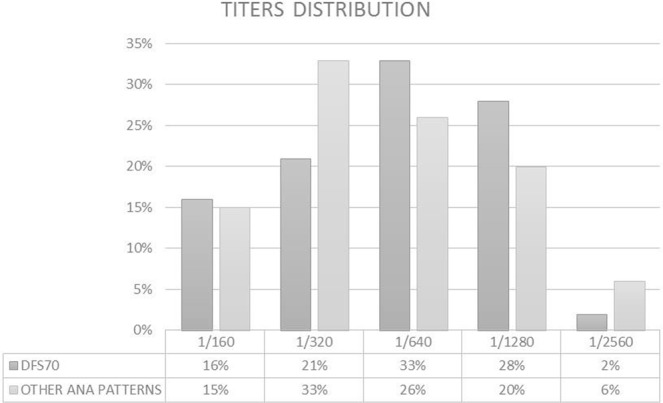
Distribution of IIF titers of anti-DFS70 antibodies and of Antinuclear Antibodies (ANA).

**Figure 4:**
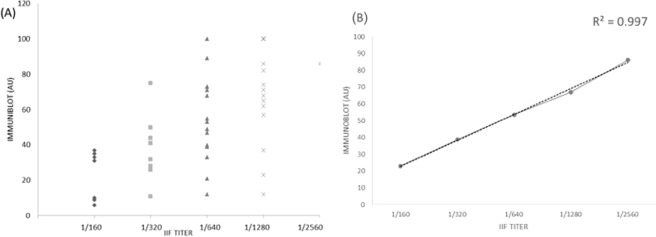
Quantitative correlation between IIF and immunoblotting. (**A**) Correlation between the anti-DFS70 antibodies concentrations by IIF titer and by immunoblotting AU. Excellent correlation between the anti-DFS70 antibodies titers by IIF (from 1:160 to 1:2560) and immunoblotting AU was found. (**B**) Regression analysis between IIF titers and quantitative immunoblotting mean values. High R^2^ value showed that the correlation has a good fit. AU: Arbitrary Unit.

Using IIF on HEp-2000 substrate at screening dilution of 1:160, 55 anti-DFS70 positive samples were identified as showing the typical DFS70 staining pattern. Titer distribution revealed no statistically differences when compared with other ANA patterns (p > 0.05). More than half of the patients presenting DFS70 monopattern (63% of the total cohort) showed high titers (≥1:640) (Fig. 3). A quantitative comparison between DFS70 IIF titers and AU immunoblotting showed an excellent correlation between two methods as expressed by a Spearman correlation (Fig. 4A). Regression analysis (R^2^ = 0.99) also confirmed the relationship between IIF titers and immunoblotting AU (Fig. 4B).

### Association of anti-DFS70 and other serum autoantibodies

**Figure 5:**
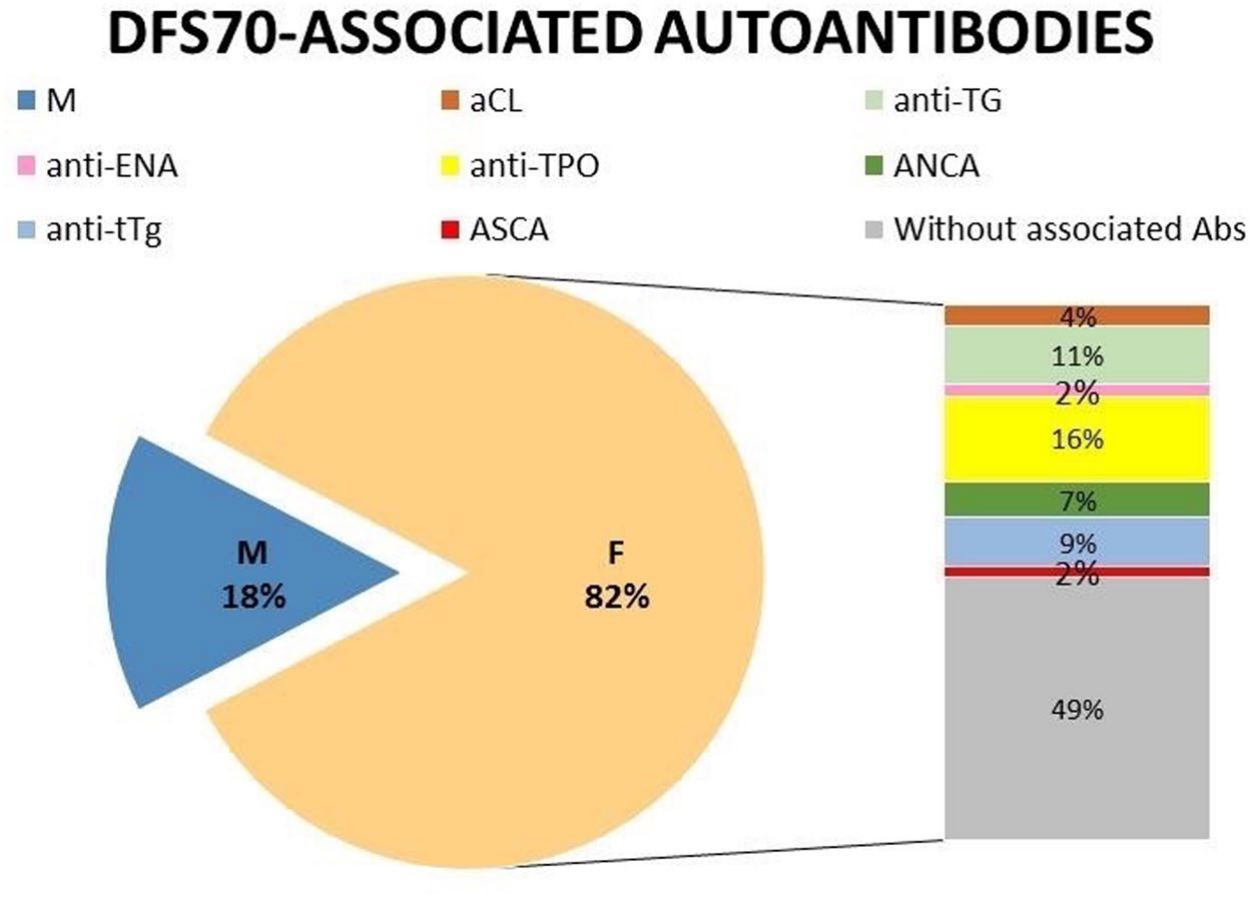
Serological features of anti-DFS70 positive patients. Concomitant of disease-related autoantibodies was found only in 51% of the anti-DFS70 antibodies positive females, no in males group. F: female, M: male. ENA: extractable nuclear antigen; TPO: thyroid peroxidase; TG, thyroglobulin; tTg, tissue transglutaminase; ASCAs, anti-Saccharomyces cerevisiae antibodies; aCL, anti-cardiolipin.

Collected DFS70-positive serum samples were assayed for a large spectrum of other circulating autoantibodies. By comparing DFS70-positive males with females, it emerged that isolated anti-DFS70 reactivity was found only in the male group. In contrast, in the 51% of DFS70-positive females, concomitant serum autoantibodies were found. Most common detected autoantibodies were anti-TPO (16.0%), anti-TG (11.0%), anti-tTg-IgA (9.0%) and ANCA (2.2% anti-PR3, 2.2% anti-MPO and 2.2% both). Only two subjects had both anti-TPO and anti-TG antibodies. ASCAs were detected only in one DFS70-positive female, also positive for anti-TPO, anti-TG and anti-tTg-IgA; anti-cardiolipin in two other and anti-ENA (anti-Mi-2 antibody) in one which also showed anti-TPO antibody positivity. No anti-DFS70 positive female, carried concomitant anti-dsDNA, anti-PCA, AMA, ASMA and anti-LKM was found (Fig. 5).

### Clinical phenotypes of anti-DFS70 positive patients

Clinical features of anti-DFS70 positive subjects were as follows: 37 healthy subjects (29 F, 8 M) and remaining (16 F, 2 M) suffered from various diseases. In the latter group 3 had spondyloarthritis (SpA), 1 morphea, 1 undifferentiated connective disease (UCTD), 3 rheumatoid arthritis (RA), 1 alopecia areata, 1 Crohn’s disease, 4 celiac disease (CD), 1 autoimmune thyroiditis (AT), 1 RA-UCTD overlap syndrome, 1 CD plus AT, and 1 SpA plus AT.

## Discussion

Previous studies using ANA-IIF test on HEp-2 substrates provide the detection of high-titer autoantibodies with typical DFS70 staining pattern in 1% to 16% of the studied cohorts. These heterogeneous results were obtained in different patient cohorts, including blood donors, healthy individuals, routine ANA cohorts, selected ANA positive healthy individuals and routine ANA positive subjects^9–22^. In details, a rate of positivity ranged from 0 to 5% was found in blood donors, healthy children and in routine ANA screening cohort. Higher frequencies were reported in ANA-positive populations. In addition, some studies examined serum samples only by IIF without specific confirmatory assays, resulting in higher rate of positivity. It has been showed that not all samples displaying DFS70 IIF pattern were then confirmed by specific CLIA or ELISA methods^23,32,33^. We analyzed 3175 consecutive unselected samples screened for ANA during the routine work-up by IIF at screening dilution of 1:160, then confirmed by immunoblotting (Fig. 1). We found 1.7% of anti-DFS70 positive sera, in line with some previous studies conducted on routine ANA screening population^6,10,13,16,19,21,34^. In fact, Mahler *et al*.^6^ analyzed 3263 sera submitted for ANA screening for the presence of anti-DFS70 antibodies and observed 1.62% of positivity. Noteworthy, Bizzaro *et al*.^15^ also reported a low frequency (0.8%) of sera displaying the anti-DFS70 reactivity on HEp-2 substrate in a large cohort of samples screened for ANA in clinical laboratory. However, because our study was performed with ANA-requested specimens from outpatients and inpatients, the overall frequency of anti-DFS70 antibodies in general population is unknown. Gender differences with greater prevalence of female (4:1) was observed in anti-DFS70 negative/ANA positive group and within the anti-DFS70 positive samples. This is an important finding since the increased immune reactivity in females predisposes them to developing AARD. A number of factors may underlie this striking gender difference and further studies are needed to better understand these findings.

Regarding referring sources of anti-DFS70 positive samples, a comparative analysis highlighted that greater prevalence of anti-DFS70 positive subjects referred from the outpatients, with value of 2.1% (Fig. 2).

We found high titers (≥1:640) in 64.3% of the DFS70 IIF-positive sera, with a titer greater than 1:2560 found only in one sample. Titer distribution revealed no statistically differences when compared with other ANA positivity (p > 0.05) (Fig. 3).

The use of specific methods to confirm DFS70 IIF suspicion was highly recommended. Recent data showed that there were no differences in diagnostic accuracy among methods using truncated (as Alphadia Dot) or full-length DFS70 antigenic sequence^34,35^. We perform a quantitative comparison among IIF titers and immunoblotting AU in anti-DFS70 positive sera resulting in excellent correlation (Fig. 4A,B).

We also analyzed the simultaneous presence of a broad spectrum of other circulating disease-markers autoantibodies. Muro *et al*., found a high prevalence of disease-related autoantibodies in anti-DFS70 positive patients, but of note, their cohort included only 22 anti-DFS70 positive females. Noteworthy, our data evidenced isolated reactivity of anti-DFS70 autoantibodies in male group, and high prevalence of disease-marker autoantibodies in females (51%). In particular, most common detected autoantibodies were anti-TPO, anti-TG, anti-tTg-IgA and ANCA (Fig. 5). It is hoped that future studies will compare the prevalence of concomitant antibodies in males versus females either in ANA positive/anti-DFS70 negative group.

Major strengths of our study are the use of anti-DFS70 confirmatory assay to avoid false positive results and the gender analysis in appraisal of the anti-DFS70 antibodies prevalence. The prevalence of anti-DFS70 was previously assessed in different cohorts mostly smaller than ours, therefore, we believe that our anti-DFS70 positive cohort should be considered suitable for a preliminary evaluation although a future larger confirmatory study is needed. In contrast, the scarce number of anti-DFS70 positive males could be considered inadequate to draw any firm conclusions. Therefore, based on low male prevalence, it is mandatory to screen larger cohort by future multicentre studies.

## Conclusion

According to previous observations performed on routine ANA cohorts, our prevalence of anti-DFS70 positivity was 1.7% of adult screened population. Serological profile of anti-DFS70 positive females required further clinical and laboratory investigations in order to define the presence of associated disease-marker autoantibodies and their clinical significance. In contrast, isolated anti-DFS70 specificity in male population suggests that the DFS70 could be considered reliable screening indicator for absence of other circulating autoantibodies resulting in considerable cost-saving potential.

## Data Availability

The dataset generated and analyzed during the present study are not publicly available, owing to restrictions by our organization, but they are available from the corresponding author on reasonable request.

## References

[1] Meroni PL, Schur PH (2010). ANA screening: an old test with new recommendations. Ann. Rheum. Dis..

[2] Leuchten N (2018). Performance of Antinuclear Antibodies for Classifyng Systemic Lupus Erithematosus: A systematic literature review and meta-regression of diagnostic data. Arthritis Care Res..

[3] Damoiseaux J (2016). International consensus on ANA patterns (ICAP): the bumpy road towards a consensus on reporting ANA results. Auto. Immun. Highlights..

[4] Ochs RL (2000). Autoantibodies to DFS 70 kd/transcription coactivator p75 in atopic dermatitis and other conditions. J. Allergy Clin. Immunol..

[5] Chan EK (2015). Report of the First International Consensus on Standardized Nomenclature of Antinuclear Antibody HEp-2 Cell Patterns 2014–2015. Front. Immunol..

[6] Mahler M (2012). Anti-DFS70/LEDGF antibodies are more prevalent in healthy individuals compared to patients with systemic autoimmune rheumatic diseases. J. Rheumatol..

[7] Ganapathy V, Casiano CA (2004). Autoimmunity to the nuclear autoantigen DFS70 (LEDGF): what exactly are the autoantibodies trying to tell us?. Arthritis Rheum..

[8] Shinohara T, Singh DP, Fatma N (2002). LEDGF, a survival factor, activates stress-related genes. Prog. Retin. Eye Res..

[9] Mahler M, Hanly JG, Fritzler MJ (2012). Importance of the dense fine speckled pattern on HEp-2 cells and anti-DFS70 antibodies for the diagnosis of systemic autoimmune diseases. Autoimmun. Rev..

[10] Lee H, Kim Y, Han K, Oh EJ (2015). Application of anti-DFS70 antibody and specific autoantibody test algorithms to patients with the dense fine speckled pattern on HEp-2 cells. Scand. J. Rheumatol..

[11] Watanabe A (2004). Anti-DFS70 antibodies in 597 healthy hospital workers. Arthritis Rheum..

[12] Vazquez-Del Mercado M (2017). Detection of autoantibodies to DSF70/LEDGFp75 in Mexican Hispanics using multiple complementary assay platforms. Auto. Immun. Highlights..

[13] Pazini AM, Fleck J, dos Santos RS, Beck ST (2010). Clinical relevance and frequency of cytoplasmic and nuclear dense fine speckled patterns observed in ANA-HEp-2. Rev. Bras. Reumatol..

[14] Schmeling H (2015). Autoantibodies to Dense Fine Speckles in Pediatric Diseases and Controls. J. Rheumatol..

[15] Bizzaro N (2007). Antibodies to the lens and cornea in anti-DFS70-positive subjects. Ann. N. Y. Acad. Sci..

[16] Bizzaro N (2015). Specific chemoluminescence and immunoasdorption tests for anti-DFS70 antibodies avoid false positive results by indirect immunofluorescence. Clin. Chim. Acta..

[17] Gundin S (2016). Measurement of anti-DFS70 antibodies in patients with ANA-associated autoimmune rheumatic diseases suspicion is cost-effective. Auto. Immun. Highlights..

[18] Mariz HA (2011). Pattern on the antinuclear antibody-HEp-2 test is a critical parameter for discriminating antinuclear antibody-positive healthy individuals and patients with autoimmune rheumatic diseases. Arthritis Rheum..

[19] Marlet, J. *et al*. Thrombophilia Associated with Anti-DFS70 Autoantibodies. *PLoS One*. **10**, 10.1371/journal.pone.0138671 (2015). 10.1371/journal.pone.0138671 PMC4580612 26397729

[20] Sperotto F, Seguso M, Gallo N, Plebani M, Zulian F (2017). Anti-DFS70 antibodies in healthy schoolchildren: A follow-up analysis. Autoimmun. Rev..

[21] Mahler, M. & Fritzler, M. J. The clinical significance of the dense fine speckled immunofluorescence pattern on HEp-2 cells for the diagnosis of systemic autoimmune diseases. *Clin. Dev. Immunol*. **2012**, 10.1155/2012/494356 (2012). 10.1155/2012/494356 PMC3523143 23304189

[22] Sener AG, Afsar I (2015). Frequency of dense fine speckled pattern in immunofluorescence screening test. Eur. J. Rheumatol..

[23] Bizzaro N, Tonutti E, Villalta D (2011). Recognizing the dense fine speckled/lens epithelium-derived growth factor/p75 pattern on HEP-2 cells: not an easy task! Comment on the article by Mariz et al. Arthritis Rheum..

[24] Bentow C, Fritzler MJ, Mummert E, Mahler M (2016). Recognition of the dense fine speckled (DFS) pattern remains challenging: results from an international internet-based survey. Auto. Immun. Highlights..

[25] Dellavance A (2005). The clinical spectrum of antinuclear antibodies associated with the nuclear dense fine speckled immunofluorescence pattern. J. Rheumatol..

[26] Ochs RL, Stein TW, Peebles CL, Gittes RF, Tan EM (1994). Autoantibodies in interstitial cystitis. J. Urol..

[27] Ochs RL (2016). The significance of autoantibodies to DFS70/LEDGFp75 in health and disease: integrating basic science with clinical understanding. Clin. Exp. Med..

[28] Daniels T (2005). Antinuclear autoantibodies in prostate cancer: immunity to LEDGF/p75, a survival protein highly expressed in prostate tumors and cleaved during apoptosis. Prostate..

[29] Muro Y, Sugiura K, Morita Y, Tomita Y (2008). High concomitance of disease marker autoantibodies in anti-DFS70/LEDGF autoantibody-positive patients with autoimmune rheumatic disease. Lupus..

[30] Seeling CA, Bauer O, Seeling HP (2016). Autoantibodies against DFS70/LEDGF wxclusion markers for systemic autoimmune rheumatic disease (SARD). Clin. Lab..

[31] Infantino, M. *et al*. Anti-DFS70 autoantibodies in undifferentiated connective tissue diseases subjects: what’s on the horizon? *Rheumatology*. 10.1093/rheumatology/Key012 (2018). 10.1093/rheumatology/key012 29618128

[32] Kang SY, Lee WI (2009). Clinical significance of dense fine speckled pattern in anti-nuclear antibody-test using indirect immunofluorescence method. Korean. J. Lab. Med..

[33] Togay A (2018). Evaluation of samples with DFS Staining Pattern Detected by Indirect Immunofluorescence assay. Clin. Lab..

[34] Carter, J. B., Carter, S., Saschenbrecker, S. & Goeckeritz, B. E. Recognition and relevance of anti- DFS70 Autoantibodies in Routine Antinuclear Autoantibodies Testing at a CommunityHospital. *Front. Med*. **5**, 10.3389/fmed.2018.00088 (2018). 10.3389/fmed.2018.00088 PMC5900435 29686987

[35] Bizzaro N (2016). Anti-DFS70 antibodies detected by Immunoblot methods: A reliable tool to confirm the dense fine speckled ANA pattern. J. Immunol. Methods.

